# Pre-service primary educators´ alternative conceptions of COVID-19 and post-COVID-19: a study of Universidad Técnica del Norte, Ecuador

**DOI:** 10.12688/f1000research.129488.1

**Published:** 2023-01-16

**Authors:** Frank Guerra-Reyes, Miguel Naranjo-Toro, Andrea Basantes-Andrade, Eric Guerra-Dávila

**Affiliations:** 1 Facultad de Educación Ciencia y Tecnología, Network Science Research Group e-CIER, Universidad Técnica del Norte, Ibarra, Ecuador, 100105, Ecuador

**Keywords:** Pandemic, COVID-19, post-COVID-19, verbal associations, pillars of education, alternative conceptions, IRaMuTeQ, Specialized Analytical Summary, ethnographic approach

## Abstract

Background: Almost at the end of 2022, the world is experiencing a relative calm after the rigors imposed by the COVID-19 pandemic. Knowing the ideas, feelings and procedures used by people in this type of unexpected events, which exceeded all established standards of educational and health actions, can help us be prepared for the possible occurrence of similar events. This study aims to determine the main alternative conceptions that pre-service teachers hold about the pandemic and the post-pandemic within the framework of the four basic pillars of Education.

Methods: A case study was conducted using an ethnographic approach. The unit of analysis was made up of 227 students from the major of Primary Education at Universidad Técnica del Norte. Two research instruments were used to collect the data: a test for verbal associations where participants can express themselves freely with hierarchical, substitution and connotative evocations; and Specialized Analytical Summary sheets to revise and organize specialized scientific texts. The data was analyzed using the IRaMuTeQ software.

Results: At first glance, what was expressed in the two contexts, both in the pandemic and in the post-pandemic, show a similar structure; however, a deeper analysis reveals different perspectives.

Conclusions: In the end, the alternative conceptions moved from expressing fear to tranquility.

## Introduction

Almost at the end of 2022, the world is experiencing a relative calm after the rigors imposed by the pandemic that, according to the World Health Organization (WHO), would be reaching its final stage. ^1^ From the academy, the media, government agencies and other areas of civil society, it has been stated that COVID-19 leaves great teachings and learning. For thinkers like Morin, Nussbaum, and Han, it was an opportunity for change. ^2^^–^^4^ However, for others such as Žižek, Cortina and Giordano, it may be diluted without leaving any change behind. ^5^^–^^7^

Concerning this issue, contemporary psychologists, ^8^ didactics and epistemologists, ^9^ as a product of their scientific findings, have concluded that, in order to generate comprehensive teaching-learning processes, it is necessary to start from the determination of alternative conceptions, some of which, on occasion, integrate perceptions, myths, misconceptions and even social representations. Therefore, this study could also be an opportunity to improve its understanding and proactively support one of the WHO’s challenges, such as evaluating misperceptions and misconceptions related to the disease caused by the coronavirus. ^10^

In fact, evaluating these conceptions represents a necessary task as they are fundamentally linked to emotional aspects and values, ^11^^,^^12^ beliefs related to processes to reduce the world population or as a divine punishment, ^13^ environmental and traditional medicine misconceptions that claim that warm weather and the use of certain local herbs are able to inhibit the replication and transmission of the virus, ^14^ anti-vaccine conspiracy theories, ^15^ contagion by mosquito bite and effectiveness of antibiotics to treat the disease, ^16^ sexist attitudes that express that women are more likely to develop the disease, ^17^ even those that associate it with the consumption of rare foods (bats and pangolins), ^18^ and an apparent immunity of young, poor and Afro-descendant people. ^19^^,^^20^

Various studies have been conducted in the fields of health and education that demonstrate the importance of knowledge, attitudes, and practices as a prerequisite for society to understand the epidemiological dynamics, as well as the success of the biosafety measures that are adopted in a certain country. ^21^^–^^23^ While argumentations have been presented in relation to the barriers imposed by inadequate communication, ^24^^,^^25^ in the same context, other studies, from a philosophical perspective, have as well raised reflections to discover experiences and values . ^26^

Despite the apparent multidisciplinary approach, it is evident that, in the global context, the studies related to the perceptions, knowledge, attitudes, and practices associated to the pandemic are still limited. ^27^^,^^28^ Although it is true that in the Ecuadorian context there are multiple investigations in the field of health care that have to do with policies and practices for patient management, it has not been possible to determine concrete and systematic experiences related to alternative conceptions of COVID-19.

Given this reality, it was necessary to address the following question: What are the main alternative conceptions related to the COVID-19 pandemic and post-pandemic, within the framework of the four basic pillars of Education, that pre-service teachers have? Being aware of how people behave in this type of unexpected events, which exceed all established standards of educational and health actions, can help us be prepared for its inevitable recurrence. On the other hand, by understanding the social dynamics in the context of a pandemic, it will increase the possibilities to improve the training processes in teachers.

## Methods

The case study was conducted using an ethnographic approach. Out of a total of 263 students from the major of Primary Education at Universidad Técnica del Norte (UTN), 227 of them participated to make up the unit of analysis in this study. UTN is a Higher Education Institution with 12,590 Ecuadorian students and 148 foreign students from different countries: Colombia, Spain, Venezuela, Cuba, Chile, United States, Italy, Germany, Belgium, Britain, Dominican Republic and Peru.

This study comprised four stages: instrument development, data collection, data analysis, and triangulation of results.

### Ethical considerations

This study was approved by the Ethics Committee of the Research Direction at Universidad Técnica del Norte (N° UTN-CI-2022-023-R). This work was carried out in accordance with the guidelines of the university code of ethics (UTN, 2012). The students who voluntarily participated in this study signed a written informed consent to guarantee the confidentiality and anonymity of the participants.

### Stage 1. Development of the instruments

Two instruments were developed: a verbal association test ^29^ and Specialized Analytical Summary (SAS) sheets.

The test was designed to determine Primary Education majors’ verbal associations about COVID-19 and post-COVID-19, within the framework of the four basic pillars of Education ^30^: learning to know (ideas, knowledge and reflections), learning to be (attitudes, emotions and values), learning to do (procedures, practices and applications), and learning to live together (harmonious coexistence and interdependence). Given that «living together» is considered as an essential characteristic of human beings, ^31^ three conceptual pairs were integrated: knowing-living together, being-living together and doing-living together. In the end, three inducing terms (ideas, feelings, and procedures) were integrated into its content so that participants could generate three free expressions with hierarchical evocations (frequency and range of appearance), of context of expression or substitution (in their own name and of others) and connotation (negative, neutral and positive).

The SAS sheets were designed as an Excel table to extract the theoretical-methodological contributions recorded in scientific articles in: WoS, Scopus, PubMed, Scielo and Taylor & Francis. Books available in print format were also included.

### Stage 2. Collection of information

The information was collected in person during June-July 2022, with a representative sample of 227 students corresponding to 86% of the total number of students from the Primary Education major at Universidad Técnica del Norte (UTN). Their ages fluctuated between 18 and 51 years, with an average age of 22 and a mode (highest frequency) of 20 years. 178 women and 49 men participated; of this group, 26 were in the first level of the major, 35 in the second, 25 in the third, 25 in the fourth, 30 in the fifth, 28 in the sixth, 25 in the seventh and 33 in the eighth level of the same major. Among the participants, 83% self-identified as mestizo, 15% as indigenous, 1% Afro-descendant, and 1% from other ethnic groups. In relation to the area of residence, 60% live in urban areas, 36% in rural areas and 4% in peripheral sectors. Regarding literature review, it was conducted with a sample of 72 scientific articles and eight books. The search terms used were: (“alternative conceptions” or “misconceptions”) and (“COVID 19” or “pandemic”) and (“students”) and (“higher education” or “college” or “university”). Searches for original scientific articles published in Spanish, English, Portuguese, and French between 2020-2022, were delimited.

### Stage 3. Data analysis

For data analysis, the IRaMuTeQ software (R interface for texts and questionnaires multidimensional analysis) was used. The software allowed to perform a hierarchical classification analysis and a factor analysis of correspondences. Initially, a data capture sheet was developed in Excel. Table 1 summarizes the data capture table.

**Table 1.** Verbal association capture table.

Informative data							COVID-19 PANDEMIC																	
							Verbal associations related to knowing-living together																	
							Personal ideas			Ideas about classmates	Ideas about family	Ideas about Covid and learning	Personal feelings			Feelings associated with classmates	Feelings associated with family	Feelings associated with Covid and learning	Personal procedures			Procedures associated with classmates	Procedures associated with family	Procedures associated with Covid and learning
Respondent	Level	Sex	Ethnicity	Age	Residence area	Province	Words	Priority	Connotation	Words	Words	Words	Words	Priority	Connotation	Words	Words	Words	Words	Priority	Connotation	Words	Words	Words
S1	First	Man	Mestizo	23	Rural	Imbabura	Illness	2	Positive	Cough	Fever	Virtual classes	Distress	2	Positive	Stress	Fear	Sadness	Self-study	2	Positive	Exercise	Hygiene	Get motivated
S2																								

The first column contains a code to identify each respondent. The successive six columns contain information related to the participants (level of education, sex, ethnic self-identification, age, area, and province of residence). Finally, the following columns are dedicated to the three verbal associations organized according to: temporality of the pandemic (before and during) and post-pandemic (now and later); then, from each of these moments, they are disaggregated based on the three inducers (ideas, feelings, and procedures). Following each inducer, the verbal associations obtained are grouped into sub-columns based on the associations themselves (personal) and those of substitution (classmates and family) to finally order them according to the rank (priority assigned or importance in the associative continuation) and its connotation (positive, negative, or neutral assessment).

Once the table was filled out, spelling errors in the conceptual corpus were corrected to avoid counting terms that could be associated as different. Then, with the use of a software, the proximity and relationships between the elements of the textual corpus were studied and then schematized in the form of word clouds, similarity trees, lexical classification dendrograms and correspondence factorial analysis matrices. Words that are represented in a larger size in the graphs, indicate a higher frequency. When the co-occurrence between words is greater, the link lines are thicker; and, in accordance with its location, the proximity and its level of belonging go to the core or to the periphery.

The software analyzed 95% of the conceptual corpus. Related to COVID-19, 8122 occurrences (words, forms, or vocabularies) appeared, 1332 different words were found and 836 with a single occurrence (hapax). With regard to post-COVID-19, 8100 occurrences emerged, 1635 different words and 1068 hapax. The analyzed content was categorized based on the three inducers (ideas, feelings, and procedures) and two time-categories were included for comparison: before (pandemic) and after (post-pandemic).

### Stage 4. Triangulation of results

Data triangulation was conducted considering three elements: results obtained from the students, findings of other researchers and understandings and interpretations of other thinkers such as philosophers, sociologists, pedagogues, anthropologists, and science communicators.

## Results and analysis

The results are presented based on the three conceptual pairs in which the four pillars of education were integrated. The findings corresponding to “knowing-living together” were outlined as word-clouds; for “being-living together”, networks of similarity were used; and for “doing-living together”, dendrograms. At the end, in order to have an overview of the alternative conceptions, the results were presented through correspondence factorial analysis matrices.

### Knowing-living together. Ideas related to COVID-19 versus post-COVID-19

**Figure 1.**
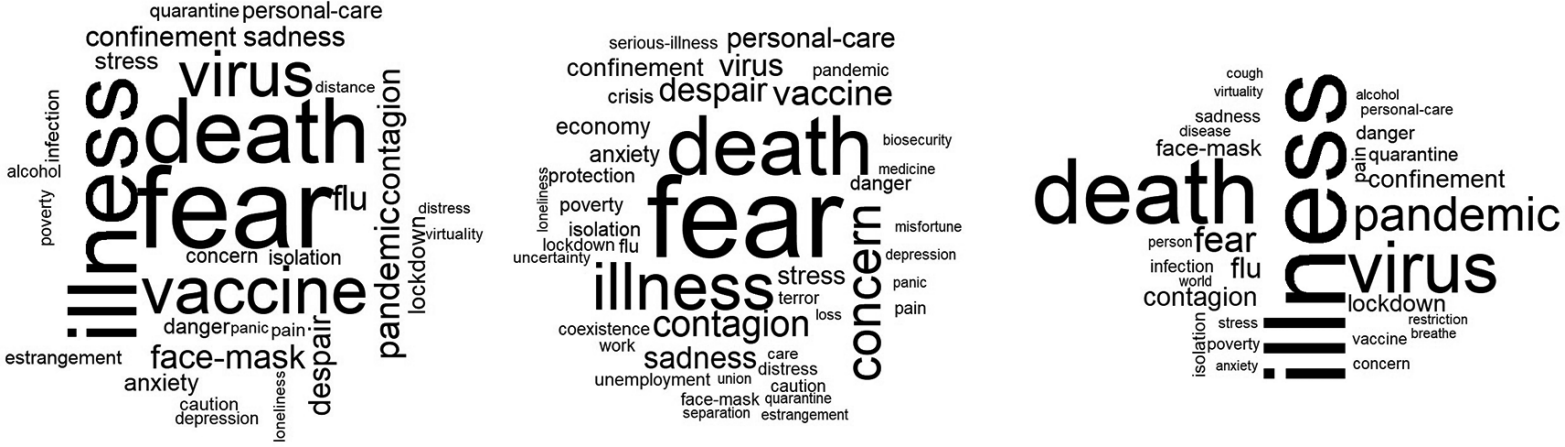
Word clouds: Ideas related to the pandemic.

In Figure 1, three word-clouds related to the category of “knowing-living together” are presented. The inducer was: ideas about the pandemic. The cloud located on the left shows the spontaneous (own) verbal associations and in the center and on the right, substitution verbal associations (classmates and family, respectively). The four words that appear most frequently were considered for the analysis. In the first cloud, words such as: illness, death, virus and pandemic stand out; in the center: fear, illness, death and vaccine; Finally, the cloud on the right show’s associations such as: fear, death, illness and concern.

As can be seen in Figure 1, the frequency and the assigned hierarchy are different when it is expressed in its own name and substitutively. If the core is reduced to two words, the results show subtle variations in conceptions. In the first instance, they revolve around the conceptual duo: illness-death; for classmates, fear-illness; and for family, fear-death. In the end, with the integration of similarity, prototypical and factorial analysis, in the three cases, a triplet (core-contrast-periphery) of the alternative conceptions was determined: as core, death; for contrast, danger; and for the periphery, quarantine.

**Figure 2.**
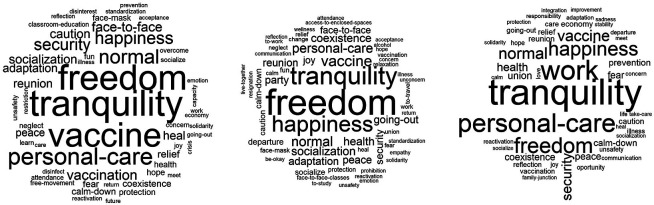
Word Clouds: Ideas related to the post-pandemic.

In relation to the “knowing-living together” dimension in the post-pandemic ( Figure 2), personal conceptions are represented in the cloud located on the left: tranquility, freedom, vaccine, and personal care; in the middle, referring to classmates: freedom, tranquility, happiness, and vaccine; and on the right, related to family: tranquility, work, personal care, and freedom. In summary, they integrate freedom-tranquility in a personal way; for classmates, freedom-tranquility; and for family, tranquility-work. In the end, due to its frequency, hierarchy and connotation, the core conception would be tranquility, learning as the contrast, and freedom on the periphery.

### Being-living together. Feelings related to COVID-19 versus post-COVID-19

**Figure 3.**
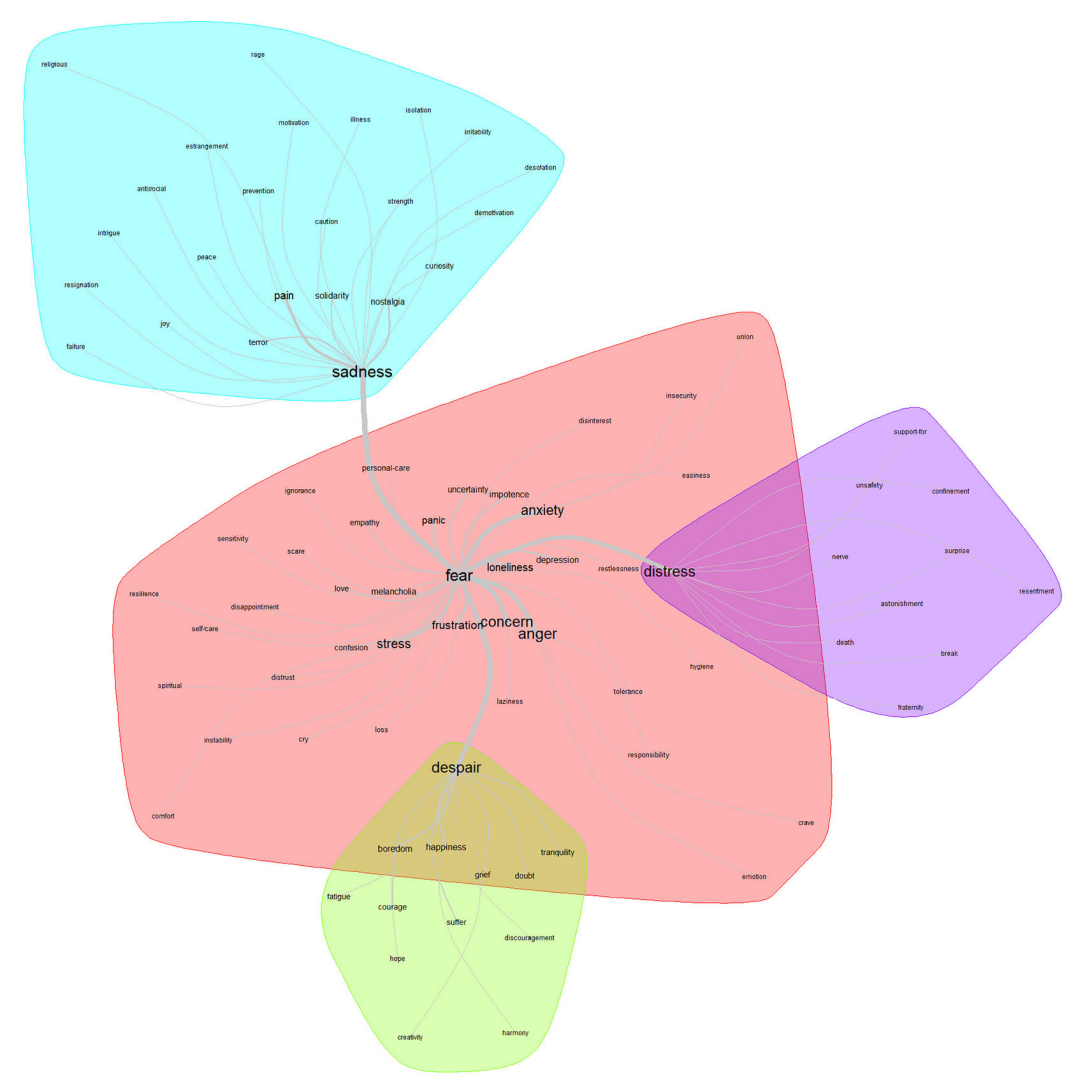
Similarity network: feelings related to the pandemic.

Figure 3 shows four lexical classes. As can be seen, “fear” appears as the core of the central class and it is strongly articulated with three more lexical classes: despair, distress, and sadness. Discursively connected with the core class: “fear”, 38 terms appear. However, linked with thicker lines, due to their semantic proximity (co-occurrence) and larger size of the words (frequency), the following are more relevant: concern, anger, anxiety, and stress. For greater specificity, based on a deeper analysis which integrates revisions of frequency and connotation, the conception that stands out as the core is fear; as the contrast, depression; and, for the periphery, loneliness.

**Figure 4.**
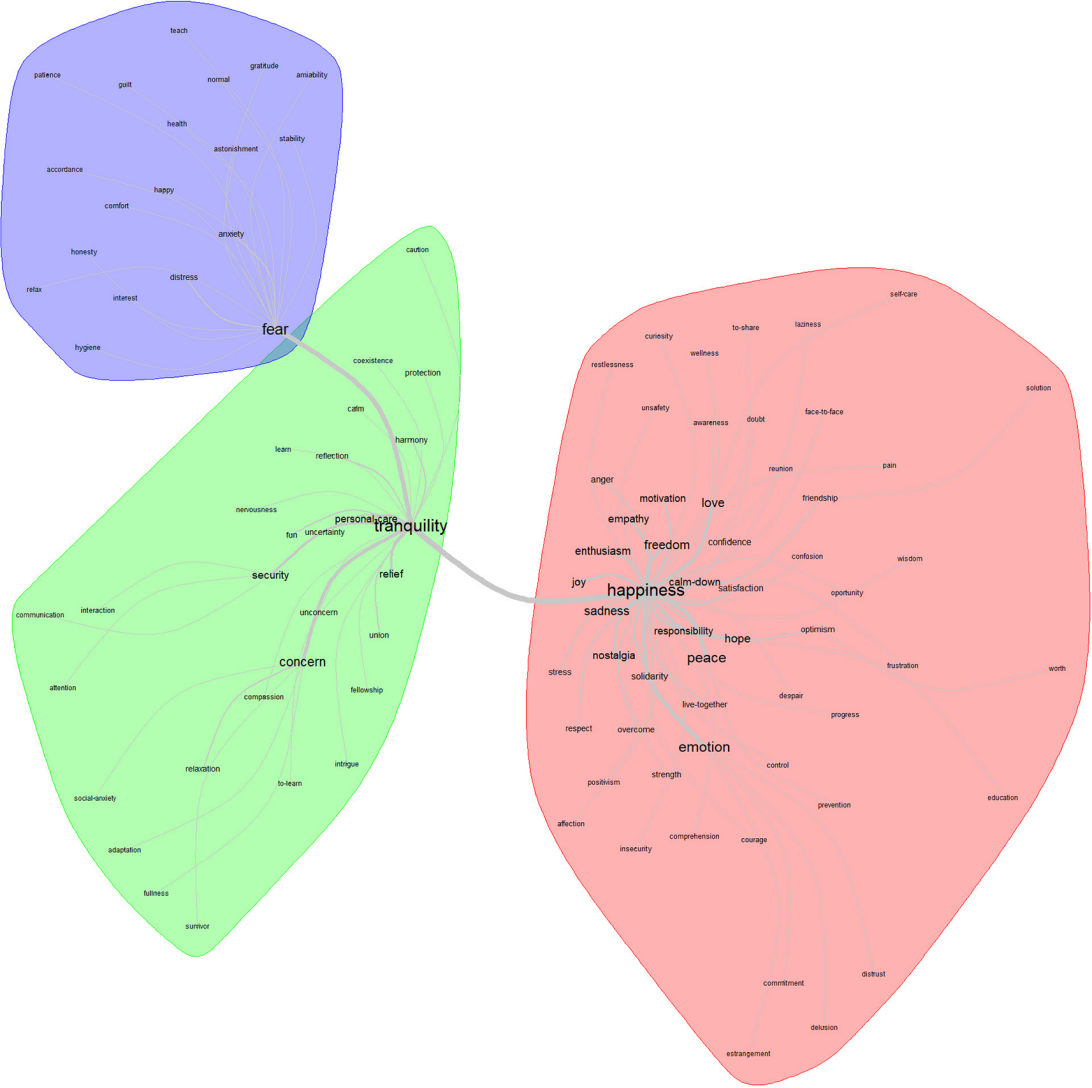
Similarity network: feelings related to the post-pandemic.

For Figure 4, the lexical class that stands out as the core in the area of feelings related to the post-pandemic is tranquility. This articulates two more classes: fear and happiness. On the periphery of the core class, 28 related terms appear.

In correspondence with the analysis procedure, the conceptions in the central class that emerge as relevant are the following: concern, personal care, security, and relief. Through the factorial analysis, the feeling that stands out as the core is “happiness”; while for contrast is empathy; and for the periphery, fear.

### Doing-living together. Procedures related to COVID-19 versus post-COVID-19

For this case, the method proposed by Reinert integrated in the IRaMuTeQ software ^32^ was used. The lemmatized conceptions (common roots or lexemes of the different variants of a word) are presented through a descending hierarchical classification table (dendrogram).

**Figure 5.**
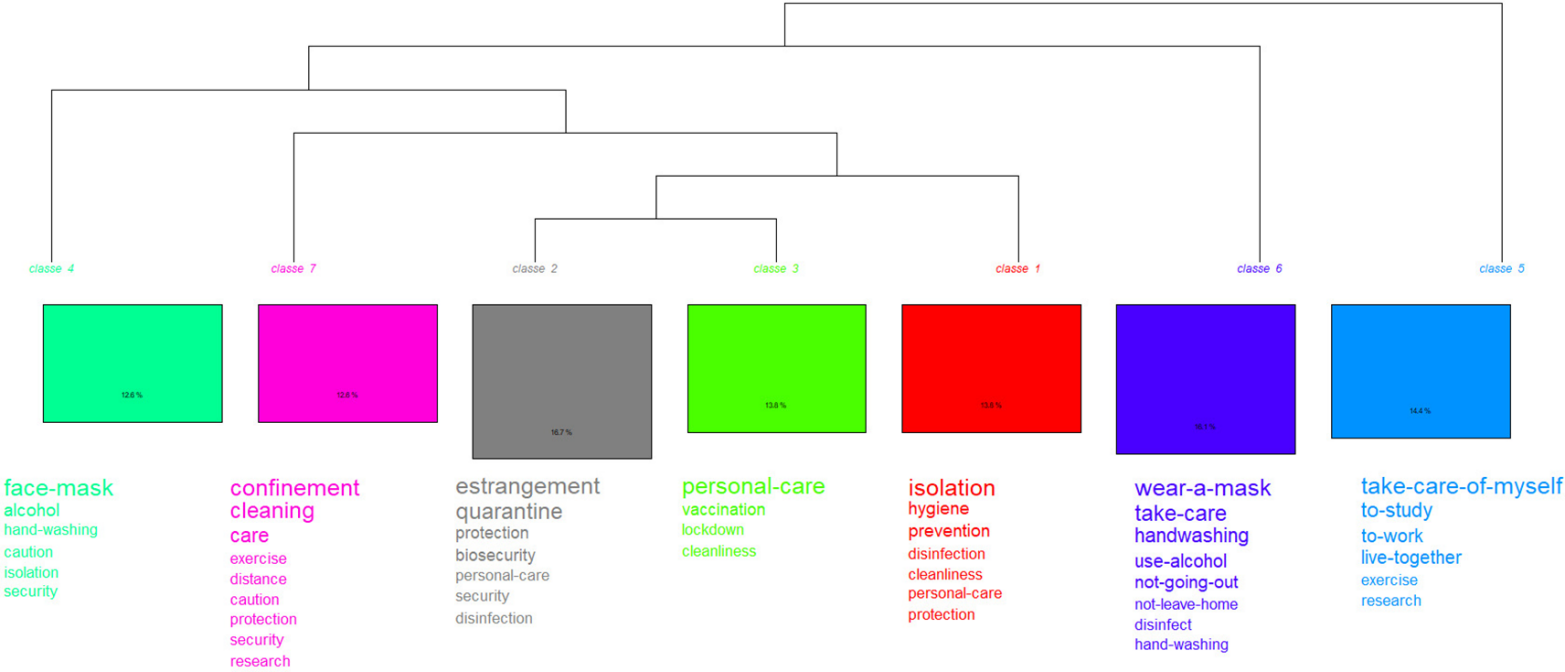
Dendrogram: procedures related to the pandemic.

In Figure 5, which shows the words with the highest connotation, two partitions (iterations) are displayed. The first one (class 5) highlights actions such as: self-care, study, work and live together, while the second one emphasizes prevention measures and resources. It is subdivided into two groups: class 6 is located on the right; and in the middle, divided into two classes: class 4 and the subgroup made up of classes {7 and [1 and (2 and 3)]}. When these results are compared with the prototypical analysis, the procedure “personal care” arises as the core; as contrast, isolation; and, on the periphery, care.

**Figure 6.**
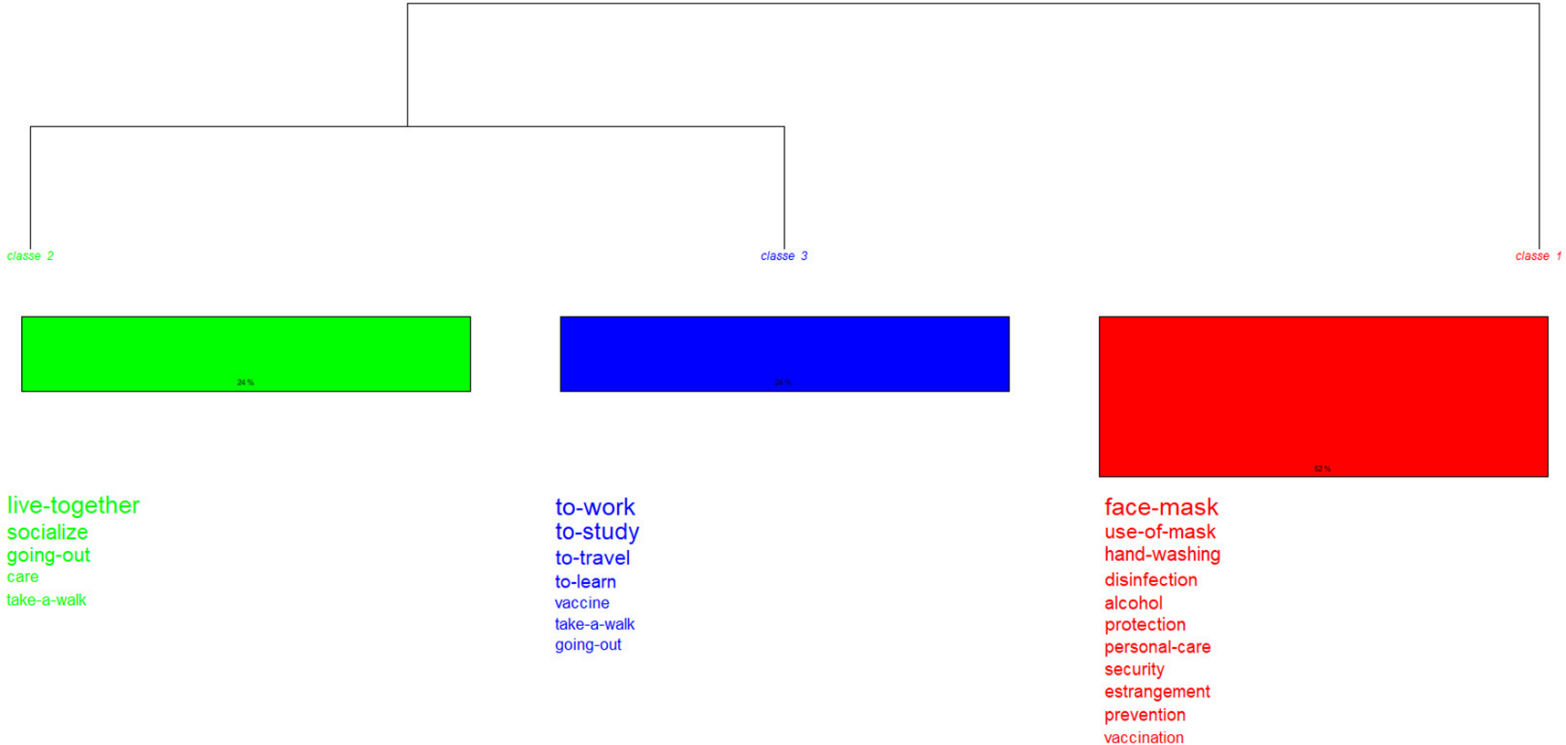
Dendrogram: procedures related to the post-pandemic.

In this dendrogram ( Figure 6) two partitions are also displayed. The first one (class 1) records biosafety measures: use of a mask, hand washing and others; the second branch, made up of classes 2 and 3, shows the procedures that became part of everyday life: living together, working, studying, and socializing. By integrating other analyses, the procedure, “personal care” appears as the core; vaccine, as contrast; and take a walk, on the periphery.

### Alternative conceptions of COVID-19: Past, present, and future

**Figure 7.**
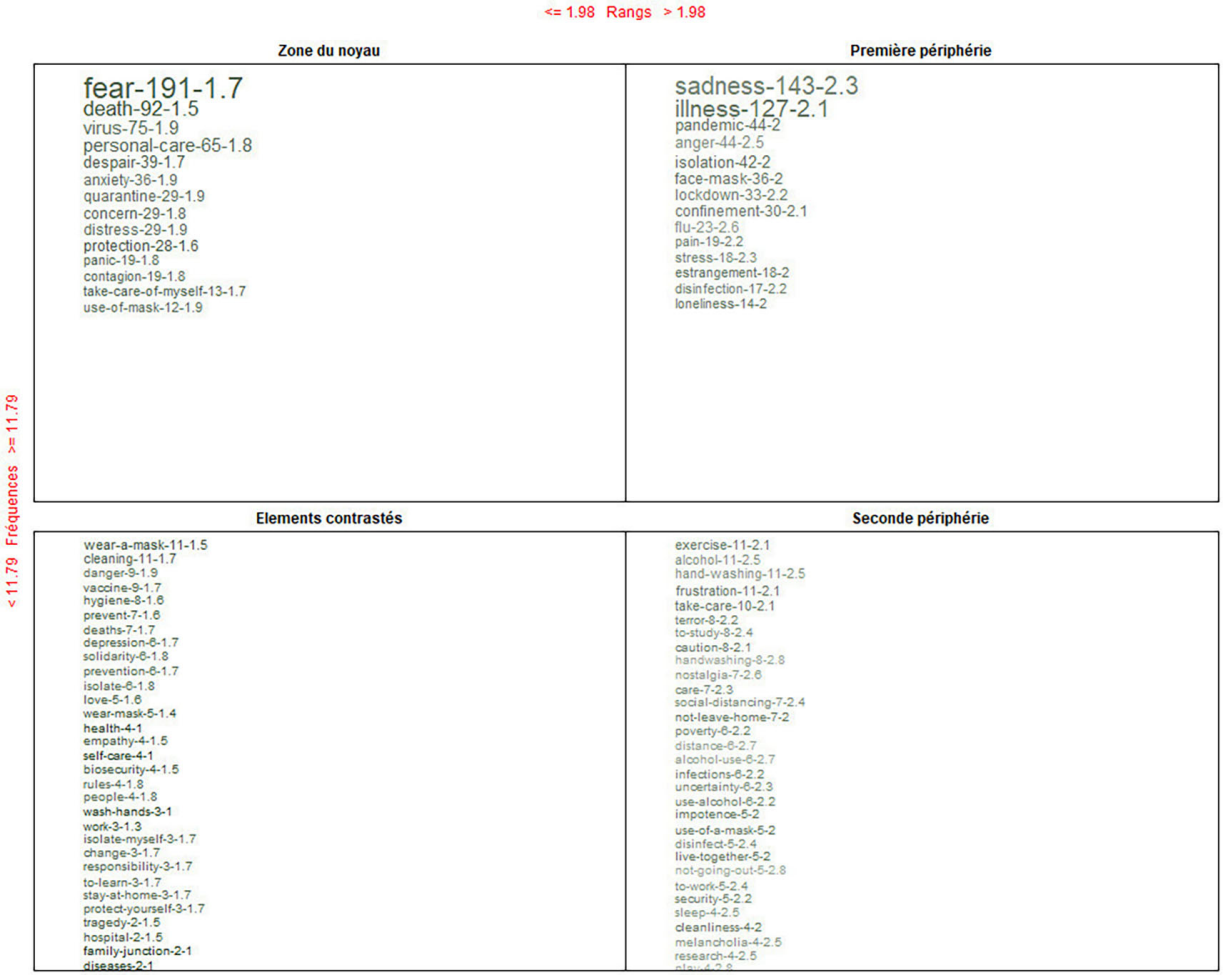
Prototypical analysis matrix: alternative conceptions of the pandemic.

Figure 7 shows the global analysis that integrates the four dimensions: knowing, being, doing and living together.

In the core zone, it is recognized that, before and during the pandemic, the dominant alternative conception is represented through the feeling “fear”; as the contrast, use of a mask; and on the periphery, exercise. When crossing the information through the Reinert method, the feeling: “fear” is connotatively consolidated.

**Figure 8.**
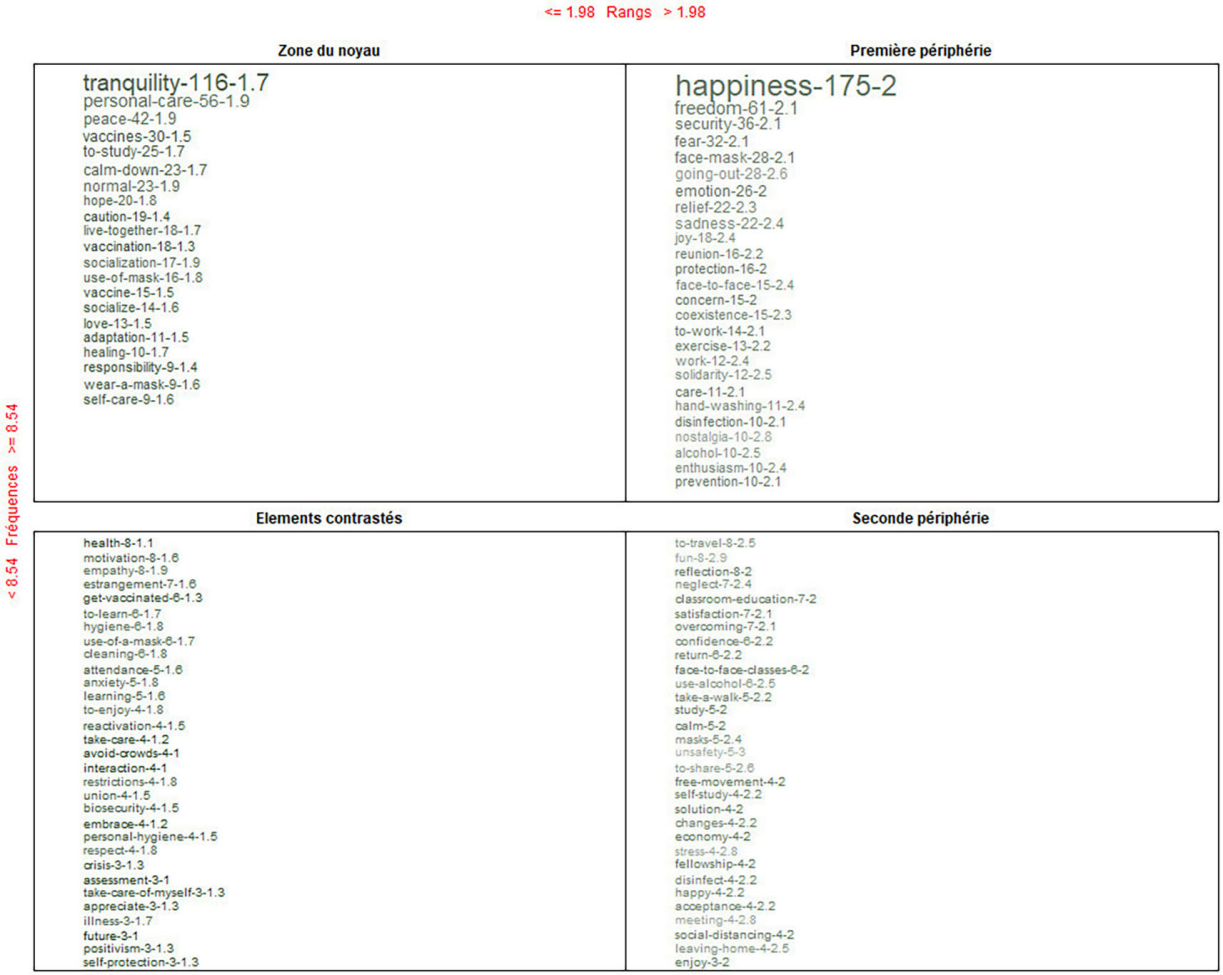
Prototypical analysis matrix: alternative conceptions of the post-pandemic.

Finally, in Figure 8, in the core area, the feeling that also stands out is “tranquility”; health, for contrast; and for the periphery, travel. With a further analysis and with the use of the Reinert method, the feeling that emerges in a dominant way is “tranquility.”

## Discussions and conclusions

Alternative conceptions are related to the common sense attributed to personal daily experiences and also to social representations of a community; hence, the interpretive validity of ideas, feelings and practices attributed individually come from there, but are also the product of the meaning assumed by a social group. ^33^^,^^34^

In this content, first, the main alternative conceptions are presented within the framework of the pillars of education. Consecutively, the interpretations determined as its conceptual core are presented: before, during and after COVID-19. For the discussion, the conceptual triplet composed of core-contrast-periphery has been prioritized.

Main alternative conceptions within the framework of the pillars of education.

Knowin-Living together. During the pandemic, the dominant ideas were: death-danger-quarantine; and, in the post-pandemic: tranquility-hope-protection.

Being-Living together. Regarding this dimension, it was the one that received the most recognition. Its triplet was as follows. In and during the pandemic, the dominant feelings were fear-depression-loneliness; and, in the post-pandemic: happiness-empathy-nostalgia.

Doing-Living together. Finally, for doing-living together, the triplets determined were, in and during the pandemic, personal care-prevention-alcohol use; and, in the post-pandemic: to study-vaccine-face-to-face classes.

In a comprehensive way, through a deeper analysis using frequency, range and connotation of the words, the dominant alternative conceptions identified were not either ideas or procedures but rather feelings. They evolved from “fear to tranquility”. Some studies coincide in locating “fear” as a core conception during COVID-19; however, they also referenced “tranquility” as its opposite in post-COVID-19. ^11^^,^^12^^,^^19^^,^^35^^,^^36^ Therefore, it is concluded that these feelings became dynamic cores of the change.

Conceptual cores of alternative conceptions: COVID-19 and POS-COVID-19.

The world is described through language, meanings are expressed, and experiences are discussed. In this sense, understandings and interpretations sustained in the context of people’s conversational activities are communicated and adapted to their social environment. ^37^ Hence, it should be considered that the alternative conceptions: “fear and tranquility” are provided with a variety of discernments of knowing, feeling, doing, and living together.

After the pandemic, “fear” acquired new meanings and even an additional feeling, cataloged as “derived fear”. ^35^^,^^38^ On the one hand, it has become something natural and collective, but also irrational, uncontrollable, contagious, and crazy. Likewise, adjectives were added to it, such as: persuasive, when it promoted awareness raising; paralyzing, when it generated impotence, insecurity, and anger (derived from gossip, sensational news); but also, adjectives like “funny”, because of the number of occurrences implemented to make it look softer, to the point that vignettes, memes and videos were and still are being spread through various social networks.

In addition, fear, as a primary emotion, generated experiences that have had an impact on knowing, feeling, doing, and living together. Fear of privacy being threatened when people requested to subscribe to government and non-government applications that could monitor and alert of possible sources of contagion. Fear as a trigger for mass and speculative consumption of items such as: face masks, alcohol, disinfectants, soap, medicines, among others.

There are different conceptions of fear in relation to the use of adjectives or qualifiers attached to it, among them: hopeful fear, understood as an opportunity for the change of paradigms in all orders of human activity; solidarity fear, coming from the multiple expressions of solidarity during the pandemic that were well evidenced in donations of economic and material resources, the massive applause for the health personnel, release of books and films, composition of songs and/or adaptation of others that were sung at all times: “I will endure”; manifestations of unity for a common purpose.

On the other hand, the fear committed to nature, focused on questioning human activities and their role in devastating the planet. In this context, many images of nature as a new free paradise with exuberant vegetation and domestic and wild fauna circulated on the web; in contrast with humans who are prisoners and are limited in their free will.

Fear was also conceived as the means for resistance and questioning the established power (current governments) due to either poor or good management of the crisis during the pandemic. The globalization of fear was felt like never before in all of humanity. Adjectives that labeled it as responsible for all misfortunes, including corruption that also had its leading role in several Latin American countries when it was about taking care of ourselves and others. ^39^^,^^40^ In the same way, the fear caused by the media and social networks that, although they helped disseminating biosecurity measures, were also responsible for spreading fake news and hoaxes.

In the end, fear was felt as a factor of distrust, which placed the blame for tragedy on the other; as a possible source of contagion and bearer of death, tragically experienced by many at the beginning of the pandemic in various parts of the planet. In this context, the algorithm of fear was created: V + D = F ^3^; that is: virus + distrust = fear tripled. ^36^

Regarding the alternative conception of transit that emerged in the post-COVID-19, “tranquility” has also been accompanied and described with some other adjectives. Tranquility as a mobilizing emotion that made people return to work and study in person, confident tranquility, for the renewed confidence and accreditation to science for its achievements in vaccines and medicines for medical treatment; empathic tranquility, for the return to interaction with peers, their support and solidarity, since they did not represent possible vectors of contagion.

Generative tranquility, due to the possibility of returning to travel, to generate new jobs and entrepreneurship ideas. Inspiring tranquility, for the work of the health personnel who risked their lives to save others. Tranquility also as a breaking point, between the perceived and lived horrors and the opportunity to continue with life with greater illusions.

Cathartic tranquility, as a passionate purification filter and liberation from painful moments that altered the minds of people and humanity having overcome death. According to Elisabeth Kübler-Ross, cited by Žižek, in tragic experiences that break into our apparent stability, one goes through at least five phases: denial, anger, negotiation, depression and acceptance. ^5^

Joyful tranquility as the experience of a state that arises from within, but also as religiosity, the return to pious experiences and recollection, from faith. Tranquility as a form of spiritual serenity after looking within and living what is essential. ^41^^,^^42^ Finally, careful tranquility, through transference, associated with sensitivity to people’s feelings, inference from other worldviews, empathy, dialogue, putting the ego in perspective and caring for the growth of others. ^43^

## Data Availability

Open Science Framework. Case_study_Alternative Conceptions of COVID-19 and post-COVID-19. DOI: 10.17605/OSF.IO/UBV6C This project contain the following underlying data:
- re-Service Primary Educators’ Alternative Conceptions of COVID-19 and post-COVID-19. A case study of Universidad Técnica del Norte. Ecuador. Data are available under the terms of the Creative Commons Attribution 4.0 International license (CC-BY 4.0).
