# Supplementary material for: Alternative conceptions of COVID-19 and post-COVID-19 assumed by Basic Education pre-service teachers. A case study for Universidad Técnica del Norte, Ecuador
Source: F1000Res. 2023 Mar 28;12:67. Originally published 2023 Jan 16. [Version 2] doi: 10.12688/f1000research.129488.2 (PMC10682601; doi:10.12688/f1000research.129488.2)
Supplement: Peer review history

# Reviewer report: Reviewer response for version 2

**DOI:** 10.5256/f1000research.145063.r167946

**Published:** 2023-11-27

**Authors:** Diego Apolo

**Affiliations:** 1 Universidad Nacional de Educación, Azogues, Ecuador

I have no more comments to make, the contributions have already been made.

Is the work clearly and accurately presented and does it cite the current literature?

Partly

If applicable, is the statistical analysis and its interpretation appropriate?

Partly

Are all the source data underlying the results available to ensure full reproducibility?

Partly

Is the study design appropriate and is the work technically sound?

Partly

Are the conclusions drawn adequately supported by the results?

Partly

Are sufficient details of methods and analysis provided to allow replication by others?

No

Reviewer Expertise:

Education, Technology, Research, Communication

I confirm that I have read this submission and believe that I have an appropriate level of expertise to confirm that it is of an acceptable scientific standard.

# Reviewer report: Reviewer response for version 1

**DOI:** 10.5256/f1000research.142175.r160373

**Published:** 2023-02-27

**Authors:** Maricarmen Caldeiro-

**Affiliations:** 1 Universidad de Santiago de Compostela, Santiago, Spain

It is considered the investigation deals with a current study event. In fact, the effects of COVID-19 have been widely studied in terms of its health implications, but very limited in the educational field. Due to its approach and the techniques used, it constitutes a different perspective of study. The verbal association technique not only allows knowing individual conceptions, it also makes it possible to determine the connotative implications of a human group, in this case of students who are trained as teachers. For these reasons, it is believed that the methodological treatment has been correct and well supported. Congratulations to the authors of the research, since it constitutes a contribution both to the theory and to the way of approaching educational studies. 

 However, with the purpose of contributing to its presentation, the following is suggested: In the body of the manuscript it is mentioned that they are students who are being trained to become basic education teachers, therefore it is suggested that this level of study be specified in the title.  In the introduction, locate a paragraph that relates studies that have also been carried out in the Latin American context and if possible in the country where the study was carried out: Ecuador  In methodology, support why it is considered to be an ethnographic study.

Is the work clearly and accurately presented and does it cite the current literature?

Yes

If applicable, is the statistical analysis and its interpretation appropriate?

Yes

Are all the source data underlying the results available to ensure full reproducibility?

Yes

Is the study design appropriate and is the work technically sound?

Yes

Are the conclusions drawn adequately supported by the results?

Yes

Are sufficient details of methods and analysis provided to allow replication by others?

Yes

Reviewer Expertise:

ICT, education

I confirm that I have read this submission and believe that I have an appropriate level of expertise to confirm that it is of an acceptable scientific standard.

# Response

**Published:** 2023-03-01

**Authors:** Andrea Basantes-Andrade

**Affiliations:** Universidad Técnica del Norte, Ecuador

Dear reviewer, we appreciate your observations, and we respond to your suggestions:

- Point 1. In the body of the manuscript it is mentioned that they are students who are being trained to become basic education teachers, therefore it is suggested that this level of study be specified in the title.

The title would read as follows:

 Alternative Conceptions of COVID-19 and post-COVID-19 assumed by Basic Education pre-service teachers. A case study for Universidad Técnica del Norte – Ecuador

- Point 2. In the introduction, locate a paragraph that relates studies that have also been carried out in the Latin American context and if possible in the country where the study was carried out: Ecuador

References to studies conducted in the Latin American and specifically in the Ecuadorian context have been integrated into the document:

 It is known that the Ibero-American Network for Research in Imaginaries and Representations (RIIR) began, in 2020, a study on the perceptions of COVID-19 social impact in the Ibero-American context. [29] Ten countries participated: Argentina, Bolivia, Brazil, Chile, Colombia, Ecuador, Spain, Mexico, Uruguay and Venezuela. The search has revealed that the research, due to its scope, is still in progress. At the Latin American level, in Mexico, through the results generated in the research named "COVID-19: Meaning and Senses," it was found that people thoughtlessly reproduced scientific, official, and even false information. [30]

 However, a systematic review is available on theses conducted and scientific articles on social representations published by two postgraduate universities: Flacso and Andina. ^31^

- Point 3. In methodology, support why it is considered to be an ethnographic study.

The research is an ethnographic study given that it is oriented to understand the alternative conceptions of COVID-19 that were assumed by the students from the major of Basic Education at Universidad Técnica del Norte, during almost two years that the pandemic lasted.

 Cordially

 Andrea Basantes-Andrade

# Reviewer report: Reviewer response for version 1

**DOI:** 10.5256/f1000research.142175.r162846

**Published:** 2023-02-21

**Authors:** Diego Apolo

**Affiliations:** 1 Universidad Nacional de Educación, Azogues, Ecuador

The issue is relevant and congratulates the proponents, this type of research provides lights for studies that deepen the theme not only at the local level but also regional. The methodological approach is very interesting but requires being based to provide the necessary support to the proposal from the basic structure: focus, techniques, instruments, analysis methods, population and/or sample.

 I allow myself to provide some suggestions that could strengthen work:

 • There are studies at the global, regional and local levels that have contributed to the subject, which are not in the introduction, it is also important to strengthen that epigraph.

 • The way in which the methodology is developed, emphasizing the category that were analyzed, as well as the way in which the instruments were built were developed.

 • The methodology requires being based from contributions from other researchers who have addressed the subject or justify the approach presented.

 • In Ecuador Primary Education is not handled; but, Basic Education with sub -levels, the title and the way of presenting it is confusing.

 • It is suggested to make a style correction for a specialist or native with academic knowledge.

 • Improve the presentation of Table 1, it is not considered adequate to present it vertically.

 • Organizing the discussion with the conclusions and not with the results hinder the understanding of the reader.

 • It is suggested to review the references manual and unify them.

 Congratulations to author or authors are reiterated, for a text that may surely be a contribution.

Is the work clearly and accurately presented and does it cite the current literature?

Partly

If applicable, is the statistical analysis and its interpretation appropriate?

Partly

Are all the source data underlying the results available to ensure full reproducibility?

Partly

Is the study design appropriate and is the work technically sound?

Partly

Are the conclusions drawn adequately supported by the results?

Partly

Are sufficient details of methods and analysis provided to allow replication by others?

No

Reviewer Expertise:

Education, Technology, Research, Communication

I confirm that I have read this submission and believe that I have an appropriate level of expertise to confirm that it is of an acceptable scientific standard, however I have significant reservations, as outlined above.

# Response

**Published:** 2023-03-01

**Authors:** Andrea Basantes-Andrade

**Affiliations:** Universidad Técnica del Norte, Ecuador

Dear reviewer, we appreciate your observations, and we respond to your suggestions through the following link:

Reviewer_response

 Cordially

 Andrea Basantes-Andrade
